# Effectiveness of video learning materials for musculoskeletal physical examinations in medical skills: a randomized controlled trial

**DOI:** 10.1186/s12909-026-09943-9

**Published:** 2026-07-17

**Authors:** Shannon Dwyer, Amalia Seibel, Hannah Chang, Marina Georgies, Rebecca Phelan, Samone Alexander, Rojin Shariflari, Chisom Nwosu, Arleigh-Ann Byer, Alexandra Hong, Denise Nadora, Puja Kulkarni, Shilpi Kalra, Layla Ali, Samuel Georgies, Jose Puglisi, Sarah Preiss-Farzanegan

**Affiliations:** 1 California Northstate University College of Medicine, 9700 W Taron Dr, Elk Grove, California 95757 USA; 2 University of California, Davis, 1 Shields Ave, Davis, California 95616 USA

**Keywords:** Medical student education, Video-based learning, Musculoskeletal physical examination

## Abstract

**Background:**

Medical students frequently report low confidence in performing musculoskeletal (MSK) physical examinations despite the high prevalence of MSK complaints in clinical practice. Traditional instructional resources have often emphasized procedural demonstrations with limited integration of anatomy and pathology. Video-based learning has emerged as a promising addition, however the optimal design for MSK education remains unclear. This study aims to evaluate the effectiveness of integrated instructional videos on medical student confidence and perceived understanding of MSK physical examinations.

**Methods:**

First year medical students enrolled in a medical skills course at an accredited US medical school were randomized into control and intervention groups across multiple MSK examination modules (spine/gait, upper limb, knee, and hip/ankle/foot). The control groups received standard preparatory materials, while the intervention groups received additional custom-designed videos that incorporated anatomy and pathological correlations. All students participated in corresponding hands-on workshops. Anonymous post-workshop surveys with Likert-style questions were administered to quantify self-reported confidence and perception, along with free response questions. Quantitative data was analyzed using the Mann-Whitney U and chi squared tests, and qualitative data was organized using Taguette Software (version 1.5.2) and chi squared.

**Results:**

The number of responses for each study section ranged from 25 to 63. Participants in the intervention group reported significantly greater confidence performing spine and gait examinations (*p* = 0.0005), along with the upper limb examinations (*p* < 0.05), with higher comfort levels in interpreting anatomy, pathology and special tests. For the hip/ankle/foot group, anatomical and special test explanations were rated higher, though overall confidence did not differ from the control group. No significant differences were observed for the knee examination. Qualitative analysis indicated that intervention videos were frequently described as helpful and comprehensive, with a statistically significant difference in anatomy content between groups.

**Conclusion:**

In this group of first-year medical students, integrated instructional videos that incorporate anatomy and pathology were associated with improved self-reported confidence and perceived understanding in specific areas of MSK physical examinations. Variation in impact may reflect differences in video length, content, or student engagement, highlighting the need for standardized instructional design materials in future studies.

**Supplementary Information:**

The online version contains supplementary material available at https://doi.org/10.1186/s12909-026-09943-9.

## Introduction

Medical students consistently report low confidence in performing musculoskeletal (MSK) physical examinations, with a study highlighting significant deficiencies in both knowledge and skill application [1]. This discomfort is reflected in Objective Structured Clinical Examination (OSCE) performance and clinical settings, where students often struggle to use physical exam findings to inform diagnostic reasoning. Despite MSK complaints being among the most common reasons for outpatient physician visits, the inconsistency and inadequacy of MSK education in U.S. medical schools continue to contribute to this gap in clinical preparedness [2–4]. This pattern was also noted by Wang et al. (2021), who surveyed course representatives from eleven medical schools in California regarding the design and content of their musculoskeletal curricula [4]. As part of this survey, course representatives were asked to rate how prepared they believed their students were to “approach/triage basic musculoskeletal conditions” on a scale of 1–5 (1 = poorly prepared, 5 = extremely prepared). The mean rating of student preparedness in MSK examination, as reported by these course representatives, was 3.4 (range 2–4) [4]. No student surveyed felt extremely prepared, and the average student felt only slightly better than average regarding their MSK training. Traditional MSK instruction has often lacked the engagement and relatability that peer-led or learner-centered approaches may provide. Emerging literature suggests that incorporating peer teaching, self-examination, and novel curricular structures can enhance student confidence and performance [5]. For instance, Yu et al. (2020) demonstrated that breaking the exam into two sessions, basic anatomy and movements followed by special tests, significantly improved student confidence [5].

There is also growing interest in refining instructional methods for MSK education, particularly in preparation for OSCE sessions. While small group learning is generally favored over large lectures, instructional format alone has not consistently improved outcomes [6]. Many traditional approaches, including multidisciplinary case presentations or simple didactics, have failed to produce significant gains in competency [7, 8]. However, multiple studies support the use of video-based instruction as an effective supplement. Videos are often preferred by students for their accessibility, ability to replay content, and ease of visualizing maneuvers, though they may fall short if they lack associated clinical reasoning and anatomical context [7, 8]. Integrating these elements, alongside careful design considerations such as instructor presence and visual consistency, has been shown to boost student engagement and understanding [9].

Despite these advancements, little attention has been paid to the optimal method of teaching specific joint exams, such as spine, gait, and knee examinations. While hands-on workshops have been shown to improve OSCE scores for knee exams [10], and students have expressed preferences for video-aided instruction, there remains a gap in research exploring how best to incorporate clinical correlations and anatomical reasoning into these formats. Similarly, spine and gait examinations are underrepresented in instructional research, though early findings suggest that videos with embedded clinical relevance are promising [8]. Ultimately, improving MSK exam instruction, through well-designed, multimodal, and learner-informed approaches, is essential to improve medical student preparedness for clinical practice and diagnostic accuracy in their future careers. The purpose of this pilot study is to evaluate the impact of integrated instructional videos in improving first-year medical students’ confidence and perception of musculoskeletal physical examinations. Our hypothesis is that videos including embedded clinical correlations to teach musculoskeletal physical examinations enhance medical students’ self-confidence in relating exam findings to anatomy, performing the physical exam, and diagnostic reasoning.

## Methods

The subjects included first-year California Northstate University College of Medicine (CNUCOM) students in the Medical Skills course who consented to participate in the study. Medical Skills is a longitudinal course spanning the two-year pre-clinical curriculum. It is designed to facilitate the development of a strong foundation in core clinical competencies, such as history taking, physical examination, diagnostic interpretation, clinical documentation, medical decision making, patient communication, and professionalism prior to clinical clerkships. Instruction methods include supervised standardized patient encounters, simulated clinical procedures, hands-on workshops, and small group training. The course is integrated with the systems-based curriculum, with weekly clinical skills aligned with the corresponding clinical presentation being studied. Each week, students are required to review preparation material prior to Medical Skills sessions. In the Musculoskeletal (MSK) block, students work with professors during workshop sessions to learn and practice physical examinations.

The students were evenly randomized into the control or intervention group for each MSK exam module. The control group received preparation materials including MacLeod’s Clinical Examination textbook, Geeky Medics videos (“Spine Examination,” “Knee Joint Examination,” and “Hip Exam”), Stanford Medicine 25 videos (“The Exam for Knee Pain” and “Ankle and Foot Exam”), and the CNUCOM Medical Skills Exam Outline documents for each MSK exam module. The intervention group received the custom video in addition to the materials received by the control group. Students in the intervention group were emailed a link to the examination video on Monday of the week of the corresponding workshop.

The intervention videos were created by second year medical students at CNUCOM with the assistance of the Medical Skills Course Director. One student depicted the role of the physician and one student or standardized patient was the standardized patient. Videos were reviewed for accuracy by the Medical Skills Course Director. The content included in the videos was a structured approach to musculoskeletal physical examination. The content was based on the MSK exam outlines for the Medical Skills course. The outlines were created from sports medicine textbooks by an experienced sports medicine physician [11–13]. The video was structured around the widely accepted MSK examination sequence, inspection, palpation, ROM, strength, and special tests, as described by Yedlinsky and Peebles (2021) [14]. It covers observational inspection (e.g., posture, symmetry, structural abnormalities, etc.), followed by palpation (assessing for tenderness, warmth, soft tissue integrity, etc.). Range of motion testing is included to identify pain and functional limitation across relevant anatomical regions. Provocative clinical tests are incorporated to aid in identification of specific pathological conditions (i.e., subacromial impingement, anterior cruciate ligament injury, etc.). The videos ranged from 10 min to 25 min depending on the MSK topic module; the upper limb module contained the largest amount of learning material and thus had the longest video).

The custom video differed from the control resources in three primary ways. First, it incorporated both normal and pathologic findings within the same examination maneuver, allowing for direct side-by-side comparison. Second, it included clinical correlations linking abnormal findings and positive special tests to their relevant pathological conditions. The control resources, by contrast, demonstrated examination techniques in isolation, without comparative normal-to-abnormal presentation or contextual clinical correlations. Finally, for most of the sections there was no existing video that covered all of the elements present in the exam outline. We created a single video that included everything students needed.

All students attended and participated in the Medical Skills workshops held on November 14, November 21, December 5, and December 12, 2024. At the Medical Skills workshop, following a live demonstration of the relevant examination by the Medical Skills Course Director, students were given the opportunity to perform the physical exams with standardized patients and under the guidance of the Medical Skills Faculty. An anonymous post-session survey was administered (link sent via email to study participants) and remained open for responses until 8:00 pm on the workshop day. This study originated as a single overarching initiative to develop supplemental musculoskeletal exam videos aligned with our institution’s exam outlines, which was subsequently divided into four parallel projects, one per module (Spine/Gait, Hip/Ankle/Foot, Knee, and Upper Limb). Each project team independently developed its instructional video and survey instrument using a shared core survey as a starting point, with adaptations made to reflect the specific anatomical region and exam covered (e.g., exam name, corresponding special tests) as well as minor refinements to item wording and scale format introduced as each team’s instrument evolved. Full surveys for each module are provided in the Supplemental Materials. The survey was developed de novo by the study team, with questions informed by the specific learning objectives of the Medical Skills curriculum, including student confidence in performing the physical exam, understanding of relevant anatomy, interpretation of special tests, and application of exam findings to differential diagnosis. Questions were structured using a combination of five-point Likert scale items to quantify self-reported confidence and perception, and open-ended free-text responses to capture qualitative feedback on the strengths and weaknesses of the preparation materials. Quantitative responses were analyzed using Mann-Whitney U and chi squared tests. Free-text responses were analyzed using Taguette (version 1.5.2), an open source qualitative coding software. Two investigators independently reviewed all responses and performed inductive thematic coding, allowing themes to emerge from the data rather than applying predefined categories. After an initial review, the investigators met to develop a consensus coding framework, which was then applied to all responses. To minimize bias, coders were assigned to review responses from a module outside their own group, for example, members of the spine and gait group coded responses from the upper limb module, for a total of four reviewers (i.e. one per group). Individual codes (e.g., “helpful,” “comprehensive,” “lacking information,” “video format issue”) were subsequently grouped into broader categories reflecting positive (“strength”) or negative (“weakness”) perceptions for each instructional domain (anatomy, special tests, and video format). These aggregated categories were then compared between study groups using chi-square tests.

## Results

Free-text responses were coded by instructional domain, including anatomy, special tests, and video format. Full coding frequencies are provided in Supplementary Table S1. For the main qualitative comparison, codes were aggregated into positive comments categorized as “strengths” and negative comments categorized as “weaknesses.” A chi-square test of independence was then used to compare the proportion of strength versus weakness codes between the control and intervention groups within each domain.

In the Anatomy domain, the intervention group had a significantly higher proportion of strength codes than the control group, 70.3% versus 39.3%, respectively, χ²(1, *N* = 65) = 5.98, *p* = 0.014. In contrast, there were no statistically significant differences between groups in the Special Tests domain, 70.4% versus 62.1%, χ²(1, *N* = 56) = 0.36, *p* = 0.55, or the Video Format domain, 69.7% versus 57.1%, χ²(1, *N* = 61) = 0.71, *p* = 0.40 (Table 1). These findings suggest that students in the intervention group more frequently described the anatomy component as a strength, while perceptions of special test explanations and video format were similar between groups.

**Table 1:** Summary of strength and weakness coding by domain and study group. Strength and weakness codes were aggregated within each domain for comparison between control and intervention groups. Percentages represent the proportion of strength codes among responses coded as either strength or weakness within each domain. NA codes and descriptive subcodes were not included in the chi-square analysis

Domain	Control Strength *n*/*N* (%)	Intervention Strength *n*/*N* (%)	χ²	df	*p*-value
Anatomy	11/28 (39.3%)	26/37 (70.3%)	5.98	1	0.014
Special Tests	18/29 (62.1%)	19/27 (70.4%)	0.36	1	0.55
Video Format	16/28 (57.1%)	23/33 (69.7%)	0.71	1	0.40

### Spine/gait likert-scale analysis

Mann-Whitney U analyses for the Spine/Gait module are summarized in Table 2. Baseline confidence did not differ significantly between the control and intervention groups, suggesting comparable pre-intervention self-reported confidence. After viewing the assigned preparatory materials, students in the intervention group reported significantly higher confidence in performing the Spine/Gait examination. The intervention group also provided significantly higher ratings for the perceived quality of the anatomy, special test, and pathology explanations, as well as greater comfort with relevant anatomy and special tests. In addition, students in the intervention group reported higher confidence performing selected examination maneuvers, including the Spurling test and straight leg raise test, and rated the video as more helpful for practice and more likely to be used again for review. There were no significant between-group differences in ratings for seeking supplemental material or in confidence using physical examination findings to guide diagnosis. (Individual figures are provided in the Supplemental Material: Fig S1-S3).

**Table 2:** Spine/Gait likert-scale survey responses by study group. Data is presented as median and interquartile range (IQR) for the control and intervention groups. Survey items were scored using a 0–5 Likert scale, with higher scores indicating more favorable responses. Between-group comparisons were performed using the Mann-Whitney U test because individual Likert-scale items were treated as ordinal data. *P*-values < 0.05 were considered statistically significant. These outcomes reflect self-reported confidence, perceived understanding, and perceived instructional quality rather than objective assessment of physical examination performance

Item	Control median (IQR) *N* = 28	Intervention median (IQR) *N* = 28	Mann-Whitney U	*p*-value
Baseline Confidence	1.0 (1.0–2.0)	1.0 (1.0–1.0)	432.0	0.3798
Confidence after	3.0 (3.0–3.25)	4.0 (3.0–4.0)	199.5	0.0006
Supplemental material	4.0 (3.0–4.0)	3.0 (2.0–4.0)	472.5	0.1757
Anatomy explanation	3.0 (3.0–3.0)	4.0 (4.0–4.0)	111.0	< 0.0001
Comfortable with Anatomy	4.0 (3.0–4.0)	4.0 (4.0–4.0)	261.0	0.0135
Comfortable with special tests	3.0 (2.75–3.25)	4.0 (3.0–4.0)	223.0	0.0035
Special test explanation	3.0 (3.0–4.0)	5.0 (4.0–5.0)	119.0	< 0.0001
Pathology explanation	3.0 (2.0–3.25)	4.0 (4.0–5.0)	143.0	< 0.0001
Confidence Spurling test	3.0 (3.0–4.0)	4.0 (4.0–5.0)	172.5	0.0001
Confidence Performing Straight Leg Raise Test	4.0 (3.0–4.0)	4.0 (4.0–5.0)	248.0	0.0116
Confidence Performing Physical Exam	4.0 (3.0–4.0)	4.5 (4.0–5.0)	189.0	0.0003
Help when practicing	4.0 (3.0–4.0)	4.0 (4.0–5.0)	181.5	0.0001
Likelihood to watch again	3.5 (2.0–4.0)	5.0 (4.0–5.0)	185.0	0.0004
Confidence using physical exams to guide differential diagnosis	3.0 (2.0–3.0)	3.0 (2.75–4.0)	286.5	0.0707

### Upper limb likert-scale analysis

Likewise, the Upper Limb module was analyzed with Mann-Whitney U (Table 3). Compared with the control group, students in the intervention group reported significantly higher confidence after viewing the assigned preparatory material and gave higher ratings for anatomy explanation, comfort with special tests, special test explanation, and pathology explanation. The intervention group also reported greater confidence using physical examination findings to guide the differential diagnosis. No significant differences were observed for supplemental material, comfort with anatomy, understanding of positive and negative tests, or likelihood of watching the videos again. Overall, the Upper Limb intervention video was associated with higher self-reported confidence and more favorable perceptions of selected instructional components, particularly those related to special tests and pathology explanations.

**Table 3:** Upper Limb Likert-Scale Survey Responses by Study Group. Values are reported as median and interquartile range (IQR) for the control and intervention groups. Survey items were scored using a 0–5 Likert scale, with higher scores indicating more favorable responses. Between-group comparisons were performed using the Mann-Whitney U test because individual Likert-scale items were treated as ordinal data. *P*-values < 0.05 were considered statistically significant. These results reflect student-reported confidence, perceived understanding, and perceived instructional quality rather than objective assessment of physical examination performance or diagnostic competence

Item	Control median (IQR) *N* = 15	Intervention median (IQR) *N* = 17	Mann-Whitney U	*p*-value
Confidence after	2.0 (2.0–3.0)	3.0 (3.0–3.0)	63.0	0.0063
Supplemental material	3.0 (2.0–3.5)	3.0 (3.0–4.0)	94.0	0.1870
Anatomy explanation	3.0 (2.0–3.0)	3.0 (3.0–4.0)	71.0	0.0208
Comfortable with Anatomy	3.0 (3.0–3.5)	3.0 (3.0–4.0)	97.0	0.1940
Comfortable with special tests	2.0 (2.0–3.0)	3.0 (3.0–4.0)	45.0	0.0008
Special test explanation	3.0 (2.0–3.5)	4.0 (4.0–4.0)	43.0	0.0007
Pathology explanation	2.0 (2.0–3.0)	3.0 (3.0–4.0)	22.5	< 0.0001
Positive and Negative test	3.0 (2.5–4.0)	3.0 (3.0–4.0)	108.5	0.4485
Likelihood to Watch again	3.0 (2.0–4.0)	4.0 (4.0–4.0)	95.5	0.1947
Confidence using physical exams to guide differential diagnosis	3.0 (2.0–3.0)	3.0 (3.0–4.0)	76.0	0.0322

### Hip/ankle/foot likert-scale analysis

Mann-Whitney U analyses for the Hip/Ankle/Foot module are summarized in Table 4. Baseline confidence, confidence after reviewing the assigned preparatory material, and ratings for supplemental material did not differ significantly between the control and intervention groups. However, students in the intervention group rated the anatomy explanation, special test explanation, and pathology explanation significantly higher than students in the control group. No significant between-group differences were observed for comfort with anatomy, comfort with special tests, confidence in performing the physical examination, use of the material as a review resource, confidence interpreting special tests, or confidence using physical examination findings to guide the differential diagnosis. Overall, the Hip/Ankle/Foot intervention video was associated with more favorable perceptions of selected instructional components, particularly explanations of anatomy, special tests, and pathology, but was not associated with significantly higher self-reported confidence in performing or interpreting the physical examination.

**Table 4:** Hip/Ankle/Foot Likert-Scale Survey Responses by Study Group. Values are reported as median and interquartile range (IQR) for the control and intervention groups. Survey items were scored using a 0–5 Likert scale, with higher scores indicating more favorable responses. Between-group comparisons were performed using the Mann-Whitney U test because individual Likert-scale items were treated as ordinal data. *P*-values < 0.05 were considered statistically significant. These findings reflect student-reported confidence, perceived understanding, and perceived instructional quality rather than objective assessment of physical examination performance, diagnostic reasoning, or clinical competence

Item	Control median (IQR) *N* = 12	Intervention median (IQR) *N* = 12	Mann-Whitney U	*p*-value
Baseline Confidence	1.0 (1.0–1.25)	1.0 (1.0–2.0)	64.5	0.6106
Confidence after	3.0 (2.75–3.25)	3.0 (3.0–4.0)	58.5	0.4052
Supplemental material	3.0 (2.0–4.0)	3.0 (2.0–3.25)	78.5	0.7194
Anatomy explanation	2.5 (2.0–3.0)	3.5 (3.0–4.0)	29.0	0.0095
Comfortable with Anatomy	3.0 (2.0–3.0)	3.0 (3.0–3.25)	48.0	0.1156
Comfortable with special tests	3.0 (2.75–3.0)	3.0 (3.0–4.0)	48.5	0.1420
Special test explanation	3.0 (2.0–3.25)	4.0 (4.0–4.0)	24.5	0.0032
Pathology explanation	2.0 (2.0–3.0)	3.0 (3.0–4.0)	34.0	0.0224
Confidence Performing Physical Exam	4.0 (3.75–4.0)	4.0 (4.0–4.0)	55.5	0.1930
Use as Review	2.5 (2.0–4.0)	4.0 (3.0–4.0)	58.0	0.4111
Confidence Interpreting Special Tests	4.0 (3.0–4.0)	4.0 (3.75–4.0)	70.0	0.9222
Confidence using physical exams to guide differential diagnosis	3.0 (3.0–4.0)	3.0 (2.75–3.25)	85.5	0.4052

### Knee likert-scale analysis

Mann-Whitney U analyses for the Knee Examination module are summarized in Table 5. No statistically significant differences were observed between the control and intervention groups for any survey item. Baseline confidence, confidence after reviewing the assigned preparatory material, ratings of supplemental material, anatomy explanation, special test explanation, and pathology explanation were similar between groups. Likewise, there were no significant between-group differences in comfort with anatomy, comfort with special tests, confidence performing the physical examination, use of the video as review material, likelihood of watching the video again, or understanding positive versus negative tests. Overall, the Knee intervention video was not associated with significantly higher self-reported confidence, perceived understanding, or perceived instructional quality compared with the control materials.

**Table 5:** Knee Examination Likert-Scale Survey Responses by Study Group. Values are reported as median and interquartile range (IQR) for the control and intervention groups. Survey items were scored using a 0–5 Likert scale, with higher scores indicating more favorable responses. Between-group comparisons were performed using the Mann-Whitney U test because individual Likert-scale items were treated as ordinal data. *P*-values < 0.05 were considered statistically significant. These findings reflect student-reported confidence, perceived understanding, and perceived instructional quality rather than objective assessment of physical examination performance, diagnostic reasoning, or clinical competence

Item	Control median (IQR) *N* = 17	Intervention median (IQR) *N* = 16	Mann-Whitney U	*p*-value
Baseline Confidence	2.0 (2.0–2.0)	2.0 (1.0–2.0)	160.5	0.3439
Confidence after	4.0 (3.0–4.0)	3.5 (3.0–4.0)	162.5	0.2765
Supplemental material	4.0 (4.0–4.0)	3.0 (2.0–4.0)	176.5	0.1126
Anatomy explanation	3.0 (2.0–4.0)	4.0 (3.0–4.0)	103.0	0.2102
Comfortable with Anatomy	4.0 (3.0–4.0)	4.0 (3.0–4.0)	139.5	0.9080
Comfortable with special tests	3.0 (2.0–4.0)	3.5 (3.0–4.0)	116.0	0.4549
Special test explanation	4.0 (3.0–4.0)	4.0 (4.0–4.0)	94.0	0.0933
Pathology explanation	3.0 (3.0–4.0)	4.0 (3.0–4.0)	103.0	0.2071
More confident	4.0 (4.0–4.0)	4.0 (3.75–4.0)	135.5	1.0000
Use as Review	4.0 (3.0–4.0)	4.0 (4.0–4.0)	108.0	0.2200
Likelihood to Watch again	4.0 (2.0–4.0)	4.0 (4.0–5.0)	109.5	0.3069
Positive vs. Negative	4.0 (3.0–4.0)	4.0 (3.0–4.0)	151.5	0.5649
Confidence Performing Physical Exam	4.0 (3.0–4.0)	4.0 (3.0–4.0)	136.0	1.0000

## Discussion

This study evaluated the impact of integrated instructional videos in improving first-year medical students’ confidence and perception of musculoskeletal physical examinations. Compared to standard course preparation materials, the intervention videos provided focused explanations of anatomical, pathological, and clinical correlations for each anatomical region. Overall, students who received the intervention video reported significantly greater confidence performing the spine and gait and upper limb examinations. Additionally, the quality of anatomical explanations, special tests, and pathological mechanisms were rated higher in the intervention group compared to the control in multiple examinations. Most of the videos available to students provided a demonstration of the exam with minimal explanations but our interventional videos provided better explanations, as shown in the post-session surveys. Similarly, Behrends et al. found that when practicing with peers, 72% of students preferred uncommented videos, which depicted the natural interaction between physician and patient, compared to 55% who preferred commented videos that broke the examination down step-by-step with narrated instruction [15]. This suggests that the more authentic, unnarrated format may better support students once they are rehearsing the exam sequence with one another, as it more closely mirrors the flow of a real clinical encounter. In contrast, our results showed increased student-perceived ability to perform the examination among those who watched our video, suggesting that explicit, structured guidance may be more valuable earlier in training, before students have internalized the examination sequence.

Together, these findings support the idea that no single video format is optimal across all stages of learning: more structured, commented videos may build initial confidence and competence, while uncommented, more naturalistic videos may become more useful later, as students consolidate and rehearse skills with peers.

The results for the knee examination survey showed no statistical significance between groups. One possible explanation for this is the smaller sample size compared to the other groups. Additionally, the knee examination was taught later in the study, and participants may have experienced response fatigue, which may have precluded them from participating in an additional survey. Yoo et al. found that only 58% of YouTube videos on knee stability tests met criteria for educational suitability, defined as adequately covering the test’s purpose and procedure, and fewer than a quarter addressed limitations or precautions of the tests [16]. Given this variability in quality among freely available resources, it is possible that the control group’s suggested videos did not consistently provide the same depth of clinical correlation and procedural detail as the videos created for this study, which may have narrowed the observed difference between groups. This underscores the importance of careful vetting of video resources for teaching the knee examination, rather than assuming all supplemental video content is comparably rigorous.

The intervention videos provided improvements to self-reported confidence and perceived explanations of anatomy and special tests; however, they did not improve confidence in guiding differential diagnosis except in the upper limb examination. One explanation is that the videos may strengthen procedural knowledge and anatomical understanding but may not be sufficient for clinical or diagnostic understanding. This is consistent with findings from Stansfield et al., who found that fourth-year medical students were not using the physical exam to inform their diagnostic reasoning, instead relying on history-taking and their overall clinical impression to guide their differential.³ Together, these findings suggest that future instructional videos should move beyond demonstrating anatomy and examination technique in isolation and instead explicitly model how exam findings are integrated into a differential diagnosis.

For example, videos could pair each special test with a brief discussion of the underlying pathology it is meant to detect, the range of conditions that could produce a similar finding, and how the result should shift or narrow the examiner’s differential.

Furthermore, this points to the importance of videos as supplementary material rather than primary instruction. This is supported by Flatt et al., who conducted a randomized controlled trial comparing face-to-face teaching alone to face-to-face teaching augmented with a custom educational video for shoulder examination [17]. While there was no significant difference in baseline scores between groups, the video-adjunct group showed a significantly greater improvement in examination performance than the face-to-face-only group. Notably, the authors explicitly concluded that electronic resources should not replace face-to-face teaching, but rather serve as a complement to it, a conclusion consistent with our own findings that the intervention videos improved confidence in specific examination components without independently building the diagnostic reasoning that is typically cultivated through direct, in-person instruction and feedback. Additionally, another study developed a web-based module to teach the hand examination and found that it was an effective tool for teaching MSK exam skills, even providing some advantages over tutor-led sessions in terms of knowledge retention [18]. Taken together, these findings suggest that instructional videos are best positioned as an adjunct that reinforces and extends in-person teaching rather than a standalone substitute, and that video-based curricula should continue to be paired with direct instruction to fully support the development of both procedural competence and diagnostic reasoning.

### Limitations

This study has several limitations. The sample sizes were relatively small and variable across the different anatomical groups. All participants were from the same institution, which may limit the generalizability to other medical curricula and institutions. Participation in the overall study declined over time, as evidenced by decreasing response rates in later anatomical groups, including the knee and hip, ankle, and foot.

Response fatigue may also have contributed to the decline in participation in later groups. Additionally, all outcomes were based on self-reported perception rather than objective performance measures on quizzes or OSCE evaluations. General participant bias may have also influenced participant responses. These limitations should be considered when interpreting the outcomes of this study and highlight the need for future studies.

### Future directions

Future research is necessary to incorporate objective measures in determining the benefit of integrated video intervention in improving medical student understanding and skill in performing musculoskeletal physical examinations. Longitudinal follow-up could also determine whether integrated videos enhance long-term retention and clinical application. Expanding this study design across multiple institutions may also help reduce participant bias and increase generalizability of results.

## Conclusion

In this cohort of first-year medical students, integrated instructional videos incorporating anatomy and pathology were associated with improved self-reported confidence and perceived understanding of MSK physical examinations. These findings are limited to student perceptions of instructional quality and self-assessed confidence, and do not directly demonstrate gains in objective examination skills, knowledge, diagnostic reasoning, or clinical competence. When used alongside hands-on instruction, such videos may serve as a scalable supplement to traditional teaching methods. Variation in impact across groups may reflect differences in video length, content complexity, or student engagement, underscoring the importance of standardized instructional design and objective outcome measures in future investigations.

## Supplementary Information

Supplementary Material 1.

Supplementary Material 2.

Supplementary Material 3.

Supplementary Material 4.

Supplementary Material 5.

Supplementary Material 6.

Supplementary Material 7.

Supplementary Material 8.

Supplementary Material 9.

Supplementary Material 10.

Supplementary Material 11.

Supplementary Material 12.

Supplementary Material 13.

Supplementary Material 14.

Supplementary Material 15.

## Data Availability

The datasets used and analysed during the current study are available from the corresponding author on reasonable request.

## Abbreviations

MSK: Musculoskeletal
OSCE: Objective Structured Clinical Examination
CNUCOM: California Northstate University College of Medicine
