# Supplementary material for: Effectiveness of video learning materials for musculoskeletal physical examinations in medical skills: a randomized controlled trial
Source: BMC Med Educ. 2026 Jul 17;26:1635. doi: 10.1186/s12909-026-09943-9 (PMC13660170; doi:10.1186/s12909-026-09943-9)
Supplement: Supplementary file 1 [file 12909_2026_9943_MOESM12_ESM.pdf]

Preparation Materials for the Knee Exam Medical Skills Session - SDSSP STUDY SURVEY (Copy)

Please complete the following survey regarding the materials you used to prepare for today's session. This survey is completely anonymous and is intended to assess your self-perceived proficiency of the physical exam of the knee. This survey will not affect your grade and will only be used for research purposes.

This survey is due on Thursday, December 5th at 11:59 PM (today!). Once submitted, our research team will enter you into a raffle with 4 prizes: 1 Littman stethoscope, 1 CNU logo backpack, 2 CNU logo sweatshirts. There are no extra steps that need to be taken on your end. The Med Skills team will hold the raffle and announce 4 winners on Monday, December 16th.

Thank you for participating in our SDSSP study and for contributing to our efforts to improve musculoskeletal education at CNUCOM!

* Required

Unique ID

You should have received this unique ID via email. If you no longer have your code, please contact Shilpi Kalra (shilpi.kalra10168@cnsu.edu) for your code.

1. Please enter your unique ID. *

2. Which most accurately describes you? Choose as many as you like. *

Note: this information will only be reported in aggregate, your individual responses are anonymous and will be kept confidential, and these data will not be used for a discriminatory purpose.

- ☐ Woman
- ☐ Man
- ☐ Non-binary/Non-conforming
- ☐ I prefer not to respond
- ☐ Other

Materials used

3. Please indicate the materials that you used to prepare for today's Medical Skills workshop session on the knee exam (select all that apply): *

- [ ] MacLeod's Clinical Examination (pages 273-276)
- [ ] The knee examination outline and PowerPoint (found on the Canvas course page)
- [ ] Geeky Medics "Knee Joint Examination - OSCE Guide" YouTube video
- [ ] Stanford Medicine 25 "The Exam for Knee Pain" YouTube video
- [ ] New M2 video made for this research project
- [ ] None of the above

Student confidence PRIOR to viewing pre-workshop assigned video(s)

This question applies to both video assignments:
(1) Geeky Medics + Stanford Medicine or (2) the newly created M2 video

4. Prior to this session, did you have any experience with the musculoskeletal knee exam? (Ex. background in athletic training, sports medicine, physical therapy, etc.) *

- ◯ Yes
- ◯ No

5. If you answered "Yes" in Question 4, please describe your experience below. *

6. How confident were you in your ability to perform this exam BEFORE reviewing the preparation material? *

	Strongly Disagree	Disagree	Neutral	Agree	Strongly Agree
I was very confident in my ability to perform the exam.	◯	◯	◯	◯	◯

Student confidence AFTER viewing pre-workshop assigned video(s)

This question applies to both video assignments:
(1) Geeky Medics + Stanford Medicine or (2) the newly created M2 video

7. How likely are you to seek/use other videos (Ex. YouTube) to supplement the pre-workshop assigned video(s)? *

	Strongly Disagree	Disagree	Neutral	Agree	Strongly Agree
I am likely to seek/use other videos to supplement the pre-workshop assigned videos.	○	○	○	○	○

8. How confident are you in you ability to perform this exam AFTER reviewing the preparation material? *

	Strongly Disagree	Disagree	Neutral	Agree	Strongly Agree
I am very confident in my ability to perform the exam.	○	○	○	○	○

Student confidence in relevant anatomy

9. How well did the pre-workshop video(s) explain the relevant anatomy? *

	Strongly Disagree	Disagree	Neutral	Agree	Strongly Agree
It explained the anatomy very well.	○	○	○	○	○

10. How comfortable are you with the anatomy of the knee, as it pertains to the physical exam? *

	Strongly Disagree	Disagree	Neutral	Agree	Strongly Agree
I am very comfortable with the anatomy of the knee.	○	○	○	○	○

11. What are the video(s) strengths in explaining the relevant anatomy?

I can identify the following on a model of the knee joint. Select all that apply (free text optional): *

- [ ] ACL: anterior cruciate ligament
- [ ] PCL: posterior cruciate ligament
- [ ] MCL: medial collateral ligament
- [ ] LCL: lateral collateral ligament
- [ ] Medial meniscus
- [ ] Lateral meniscus
- [ ] Femur
- [ ] Tibia
- [ ] Fibula
- [ ] Patella
- [ ] Quadriceps tendon
- [ ] Patellar ligament
- [ ] Other

12. What are the video(s) weaknesses in explaining the relevant anatomy?

I cannot identify the following on a model of the knee joint. Select all that apply (free text optional): *

- [ ] ACL: anterior cruciate ligament
- [ ] PCL: posterior cruciate ligament
- [ ] MCL: medial collateral ligament
- [ ] LCL: lateral collateral ligament
- [ ] Medial meniscus
- [ ] Lateral meniscus
- [ ] Femur
- [ ] Tibia
- [ ] Fibula
- [ ] Patella
- [ ] Quadriceps tendon
- [ ] Patellar ligament
- [ ] Other

Attention Check

13. Please select "Disagree" if you are following along! *

Strongly Disagree	Disagree	Neutral	Agree	Strongly Agree
○	○	○	○	○

Student confidence in performing special tests

14. How confident did you feel performing the knee special tests (Apprehension test, Anterior drawer, Posterior drawer, Lachman, Varus stress, Valgus stress, McMurray) after reviewing the preparation materials? *

	Strongly Disagree	Disagree	Neutral	Agree	Strongly Agree
I was confident in performing the knee special tests during the workshop session.	○	○	○	○	○

15. How clearly did the pre-workshop assigned materials explain the special tests? *

	Strongly Disagree	Disagree	Neutral	Agree	Strongly Agree
The pre-workshop assigned materials explained the special tests clearly.	○	○	○	○	○

16. How clearly did the pre-workshop assigned materials explain the pathological mechanisms as they relate to the special tests? *

	Strongly Disagree	Disagree	Neutral	Agree	Strongly Agree
The pre-workshop assigned materials explained the pathological mechanisms clearly.	○	○	○	○	○

Student understanding of the physical exam

17. The pre-workshop assigned materials gave me more confidence in my ability to perform the knee exam. *

	Strongly disagree	Disagree	Neutral	Agree	Strongly agree
I am confident in my ability to perform the knee exam.	○	○	○	○	○

18. The pre-workshop assigned materials helped me when practicing the knee exam. *

	Strongly disagree	Disagree	Neutral	Agree	Strongly agree
I thought reviewing the pre-workshop materials helped me while practicing the knee exam.	○	○	○	○	○

19. How likely are you to watch the pre-workshop assigned materials again to review this exam? (as opposed to other supplemental materials) *

	Strongly disagree	Disagree	Neutral	Agree	Strongly Agree
I will likely watch the pre-workshop assigned materials again to review the exam.	○	○	○	○	○

Student confidence in using physical exam findings in differential diagnosis

20. How confident are you in determining a **positive special test versus a negative special test** as they pertain to this exam? *

	Strongly Disagree	Disagree	Neutral	Agree	Strongly Agree
I am confident that I am able to determine a positive special test vs. a negative special test.	◯	◯	◯	◯	◯

21. How confident do you feel using **physical exam findings** to guide your differential diagnosis (i.e., in your H&P note)? *

	Strongly Disagree	Disagree	Neutral	Agree	Strongly agree
I am confident in using the physical exam findings to guide my differential diagnosis.	◯	◯	◯	◯	◯

Evaluation of the pre-workshop assigned video(s)

This question applies to both video assignments:
(1) Geeky Medics + Stanford Medicine or (2) the newly created M2 video

22. What are the **strengths** of the pre-workshop assigned materials? *

Select all that apply.

- [ ] Content was clear and effective.
- [ ] The video(s) improved my proficiency of the knee exam.
- [ ] The materials taught me what order to perform the exam in, or helped me establish my own flow with the exam.
- [ ] The materials helped me understand the Med Skills workshop thoroughly.
- [ ] The materials reduced any potential uncertainty or nervousness going into the Med Skills workshop.
- [ ] Other

23. What can be **improved** in the pre-workshop assigned materials? *

Select all that apply.

- [ ] Content was unclear and ineffective.
- [ ] The video(s) did not improve my proficiency of the knee exam.
- [ ] Hand placement performing special tests was unclear.
- [ ] Anatomical and clinical correlations were lacking.
- [ ] The materials did not teach me what order to perform the exam in or help me establish my own flow with the exam.
- [ ] I was still confused about the knee exam going into the Med Skills workshop.
- [ ] The materials did not reduce any potential uncertainty or nervousness going into the Med Skills workshop.
- [ ] Other

24. What worked **well in the format** of the pre-workshop assigned materials? *

Select all that apply.

- [ ] Formatting of the content was clear and effective.
- [ ] Video angles allowed for clear comprehension of the exam.
- [ ] The formatting of the content improved my learning of the knee exam.
- [ ] The editing style used was sufficient.
- [ ] The quality of the audio and visual was sufficient.
- [ ] Other

25. What could be **improved in the format** of the pre-workshop assigned materials? *

Select all that apply.

- [ ] Formatting of the content was unclear and confusing at times.
- [ ] Video angles were confusing.
- [ ] The editing style of the video was confusing.
- [ ] The pacing of the material provided was too fast or too slow.
- [ ] The length of the video was not sufficient (too short or too long).
- [ ] Other

26. What worked **well in the content** of the pre-workshop assigned materials? *

Select all that apply.

- [ ] Anatomical areas were clearly defined.
- [ ] There was thorough clinical correlation to explain each exam.
- [ ] All aspects of the video were relevant to understand how to perform the knee exam.
- [ ] Other

27. What could be **improved in the content** of the pre-workshop assigned materials? *

Select all that apply:

- [ ] Anatomical areas were not clearly defined
- [ ] Clinical correlates were lacking
- [ ] Aspects of the video(s) were irrelevant to my
- [ ] The order of the content taught was confusing
- [ ] Other

This content is neither created nor endorsed by Microsoft. The data you submit will be sent to the form owner.

Microsoft Forms
