# Supplementary material for: Effectiveness of video learning materials for musculoskeletal physical examinations in medical skills: a randomized controlled trial
Source: BMC Med Educ. 2026 Jul 17;26:1635. doi: 10.1186/s12909-026-09943-9 (PMC13660170; doi:10.1186/s12909-026-09943-9)
Supplement: Supplementary file 2 [file 12909_2026_9943_MOESM13_ESM.pdf]

Preparation Materials for the Upper Limb Medical Skills Session - SDSSP STUDY SURVEY

Please complete the following survey regarding the materials you used to prepare for today's session. **This survey is completely anonymous.**
Once you have submitted the survey, you are automatically entered into the Raffle for the Littman stethoscope!
Thank you for participating in our SDSSP study and for contributing to our efforts to improve musculoskeletal education at CNUCOM!

* Required

Participant Number

1. What is your participant number? *

2. What is your age? *

3. Which most accurately describes you? Choose as many as you like. [Note: this information will only be reported in aggregate, your individual responses are anonymous and will be kept confidential, and these data will not be used for a discriminatory purpose.] *

- ☐ Woman
- ☐ Man
- ☐ Non-binary/Non-conforming
- ☐ Let me type... (please see next question)
- ☐ I prefer not to respond

4. Which most accurately describes you? Choose as many as you like. [Note: this information will only be reported in aggregate, your individual responses are anonymous and will be kept confidential, and these data will not be used for a discriminatory purpose.] *

- [ ] Woman
- [ ] Man
- [ ] Non-binary/Non-conforming
- [ ] Let me type... (please see next question)
- [ ] I prefer not to respond

5. If you selected, "Let me type...." to the previous question, please use the box below to complete your answer. (If you did not select that choice, please skip this question and proceed with the survey, thank you.)

Verification of Assignment Completion

6. **Did you watch the pre-classroom assigned video for the upper limb physical examination?** *

- Yes
- No

Pre-Classroom Video Effectiveness

Please complete the following survey regarding the effectiveness of the pre-classroom assigned videos for preparing you for today's med skills session and OSCE.

7. **Who was the "examiner" in the video series you watched?** *

◯ Geeky Medics

◯ Stanford 25

◯ CNUCOM Medical Students

8. **Did you find it helpful to split the pre-classroom assigned videos into a "look, feel, move"/anatomy portion and a special test portion?** *

◯ Yes

◯ No

◯ Somewhat

9. **Do you have experience with the upper extremity MSK exam prior to watching the videos (i.e. background in athletic training, sports medicine, etc.)?** *

◯ Yes

◯ No

10. **If you answered "yes" to the previous question, how confident did you feel in your ability to perform this exam prior to watching the pre-classroom assigned videos?**

1 - Not confident at all	2 - Slightly confident	3 - Moderately confident	4 - Very confident	5 - Extremely confident
◯	◯	◯	◯	◯

11. **How well do you feel the videos prepared you for today's med skills session?** *

1 - Very poorly	2 - Poorly	3 - Neutral	4 - Well	5 - Very well
◯	◯	◯	◯	◯

12. **How confident were you in your ability to perform the upper extremity exam after watching the pre-classroom assigned videos?** *

1 - Not confident at all	2 - Slightly confident	3 - Moderately confident	4 - Very confident	5 - Extremely confident
◯	◯	◯	◯	◯

13. **How likely are you to seek other videos (i.e. Youtube) to supplement the pre-classroom assigned video?** *

1 - Extremely unlikely	2 - Unlikely	3 - Neutral	4 - Likely	5 - Extremely likely
◯	◯	◯	◯	◯

14. **How well do you feel the pre-classroom assigned videos explained the relevant anatomy?** *

1 - Poor	2 - Fair	3 - Good	4 - Very Good	5 - Excellent
◯	◯	◯	◯	◯

15. **How comfortable are you with the upper extremity anatomy, as it pertains to the physical exam?** *

1 - Extremely uncomfortable	2 - Uncomfortable	3 - Slightly comfortable	4 - Very comfortable	5 - Extremely Comfortable
◯	◯	◯	◯	◯

16. **Please explain the video's strengths in helping explain the relevant anatomy. Did the pre-classroom videos help clarify these ideas?** *

17. **Please explain the video's weaknesses in helping explain the relevant anatomy. Is the video missing anything that would make it better?** *

18. **How confident did you feel in performing the upper extremity special tests after watching the pre-classroom assigned videos?** *

1 - Not confident at all	2 - Slightly confident	3 - Moderately confident	4 - Very confident	5 - Extremely confident
◯	◯	◯	◯	◯

19. **How well do you feel the pre-classroom assigned videos explained the special tests?** *

1 - Poor	2 - Fair	3 - Good	4 - Very Good	5 - Excellent
◯	◯	◯	◯	◯

20. **How well do you feel the pre-classroom assigned videos explained the pathological mechanisms as they relate to the special tests?** *

1 - Poor	2 - Fair	3 - Good	4 - Very Good	5 - Excellent
◯	◯	◯	◯	◯

21. **Please explain the strengths of the pre-classroom assigned videos in preparing you to perform the special tests.** *

22. **Please explain the weaknesses of the pre-classroom assigned videos in preparing you to perform the special tests.** *

23. **How likely are you to rewatch the pre-classroom assigned videos to review the upper extremity physical exam (as opposed to other supplemental videos)?** *

1 - Very unlikely	2 - Unlikely	3 - Neutral	4 - Likely	5 - Very likely
◯	◯	◯	◯	◯

24. **What worked well in the format of the pre-classroom assigned videos?** *

25. **What could be improved in the format of the pre-classroom assigned videos?** *

26. **How confident are you in determining a positive special test versus a negative special test (as they pertain to the upper extremity physical exam)?** *

1 - Not confident at all	2 - Sightly confident	3 - Moderately confident	4 - Very confident	5 - Extremely confident
○	○	○	○	○

27. **How confident do you feel in using the physical exam findings to guide your differential diagnosis (i.e. in your OSCE note)?** *

1 - Not confident at all	2 - Slightly confident	3 - Moderately confident	4 - Very confident	5 - Extremely confident
○	○	○	○	○

This content is neither created nor endorsed by Microsoft. The data you submit will be sent to the form owner.

Microsoft Forms
