# Supplementary material for: Effectiveness of video learning materials for musculoskeletal physical examinations in medical skills: a randomized controlled trial
Source: BMC Med Educ. 2026 Jul 17;26:1635. doi: 10.1186/s12909-026-09943-9 (PMC13660170; doi:10.1186/s12909-026-09943-9)
Supplement: Supplementary file 3 [file 12909_2026_9943_MOESM14_ESM.pdf]

Preparation Materials for the Spine and Gait Medical Skills Session - SDSSP STUDY SURVEY

Please complete the following survey regarding the materials you used to prepare for today's session. This survey is completely anonymous.
Your survey submission with your participant ID code will enter you in the raffle for a Littman III stethoscope!!
Thank you for participating in our SDSSP study and for contributing to our efforts to improve musculoskeletal education at CNUCOM!

* Required

1. Please enter your participant ID: *

2. Please indicate the material that you used to prepare for today's Medical Skills session on the Spine & Gait exam (select all that apply): *

- [ ] Reading assignment in MacLeod
- [ ] Geeky Medics Videos
- [ ] Our Experimental video

3. Prior to this session, did you have any experience with the Spine & Gait musculoskeletal exam? (for example, background in athletic training, sports medicine, physical therapy, etc.) *

- ◯ Yes
- ◯ No

4. If yes, please describe your experience (if no, please enter "n/a"). *

5. How confident were you in your ability to perform this exam **BEFORE** reviewing the preparation materials? *

Not confident at all	Slightly confident	Somewhat confident	Fairly confident	Completely confident
◯	◯	◯	◯	◯

6. How confident were you in your ability to perform this exam **AFTER** reviewing the preparation materials? *

Not confident at all	Slightly confident	Somewhat confident	Fairly confident	Completely confident
◯	◯	◯	◯	◯

7. How likely are you to seek/use other videos to supplement the pre-classroom assigned video(s)? *

Very likely	Somewhat likely	Neither likely nor unlikely	Somewhat unlikely	Very unlikely
◯	◯	◯	◯	◯

8. How well did the pre-classroom video(s) explain the relevant anatomy? *

Above average	Very well	Average	Below average	Not so well
◯	◯	◯	◯	◯

9. How comfortable are you with the Spine & Gait anatomy, as it pertains to the physical exam? *

Very comfortable	Somewhat comfortable	Neither comfortable nor uncomfortable	Somewhat uncomfortable	Very uncomfortable
◯	◯	◯	◯	◯

10. What are the video's **strengths** in explaining the relevant anatomy? *

11. How confident did you feel performing the Spine & Gait special tests after reviewing the preparation materials? *

Not confident at all	Slightly confident	Somewhat confident	Fairly confident	Completely confident
◯	◯	◯	◯	◯

12. How clearly did the pre-classroom assigned materials explain the special tests? *

Excellent	Good	Fair	Poor	Very poorly
◯	◯	◯	◯	◯

13. How clearly did the pre-classroom assigned materials explain the pathological mechanisms as the relate to the special tests (Spurling's test and Straight leg raise)? *

	Excellent	Good	Fair	Poor	Very poorly
Statement 1	◯	◯	◯	◯	◯

14. What are the **strengths** of the pre-classroom assigned materials in preparing you to perform the special tests (Spurling's test and Straight leg raise)? *

15. What are the **weaknesses** of the pre-classroom assigned materials in preparing you to perform the special tests (Spurling's test and Straight leg raise)? *

16. How confident are you in determining a positive Spurling's test versus a negative Spurling's test? *

Not confident at all	Slightly confident	Somewhat confident	Fairly confident	Completely confident
◯	◯	◯	◯	◯

17. How confident are you in determining a positive straight leg raise versus a negative straight leg raise as they pertain to this exam? *

Not confident at all	Slightly confident	Somewhat confident	Fairly confident	Completely confident
◯	◯	◯	◯	◯

18. The pre-classroom assigned materials gave me more confidence in my ability to perform the Spine & Gait exam *

Strongly agree	Agree	Neither agree nor disagree	Disagree	Strongly disagree
◯	◯	◯	◯	◯

19. The pre-classroom assigned materials helped me when practicing the Spine & Gait exam. *

Strongly agree	Agree	Neither agree nor disagree	Disagree	Strongly disagree
○	○	○	○	○

20. How likely are you to watch the pre-classroom assigned materials again to review this exam? (as opposed to other supplemental materials) *

Very likely	Somewhat likely	Neither likely nor unlikely	Somewhat unlikely	Very unlikely
○	○	○	○	○

21. How confident do you feel using physical exam findings to guide your differential diagnosis (i.e., in your H&P note)? *

Not confident at all	Slightly confident	Somewhat confident	Fairly confident	Completely confident
○	○	○	○	○

22. What are the **strengths** of the pre-classroom assigned materials ? *

23. What can be **improved** in the pre-classroom assigned materials ? *

24. What worked well in the **format** of the pre-classroom assigned materials? *

25. What could be improved in the **format** of the pre-classroom assigned materials? *

26. What worked well in the **content** of the pre-classroom assigned materials? *

27. What could be improved in the **content** of the pre-classroom assigned materials? *

This content is neither created nor endorsed by Microsoft. The data you submit will be sent to the form owner.

Microsoft Forms
