# Supplementary material for: Effectiveness of video learning materials for musculoskeletal physical examinations in medical skills: a randomized controlled trial
Source: BMC Med Educ. 2026 Jul 17;26:1635. doi: 10.1186/s12909-026-09943-9 (PMC13660170; doi:10.1186/s12909-026-09943-9)
Supplement: Supplementary file 4 [file 12909_2026_9943_MOESM15_ESM.pdf]

Preparation Materials for the Hip, Ankle/Foot Medical Skills Session - SDSSP STUDY SURVEY

Please complete the following survey regarding the materials you used to prepare for today's session. **This survey is completely anonymous.** Once you have submitted the survey, you are automatically entered into the Raffle for the Littman stethoscope! Thank you for participating in our SDSSP study and for contributing to our efforts to improve musculoskeletal education at CNUCOM!

* Required

Demographics

1. Enter your participant ID number *

2. What is your age? *

3. Which most accurately describes you? Choose as many as you like. [Note: this information will only be reported in aggregate, your individual responses are anonymous and will be kept confidential, and these data will not be used for a discriminatory purpose.] *

- ☐ Woman
- ☐ Man
- ☐ Non-binary/Non-conforming
- ☐ Let me type... (see next question)
- ☐ I prefer not to respond

4. If you selected, "Let me type...." to the previous question, please use the box below to complete your answer. (If you did not select that choice, please skip this question and proceed with the survey, thank you.)

Student Confidence Prior to Viewing Pre-Classroom Assigned Videos

5. Please indicate the material that you used to prepare for today's Medical Skills session on the Hip, Ankle, and Foot Exam (select all the apply) *

- ☐ Reading Assignment in MacLeod
- ☐ Video Clips
- ☐ Hip, Ankle and Foot Experimental Video

6. Prior to this session, did you have any experience with the Hip, Ankle, and Foot Musculoskeletal Exam? (Ex: background in athletic training, sports medicine, physical therapy, etc) *

- ◯ Yes
- ◯ No

7. If yes, please describe your experience. *

8. How confident were you in your ability to perform this exam BEFORE reviewing the preparation materials? *

Not Confident	Slightly confident	Moderately Confident	Very Confident	Extremely Confident
◯	◯	◯	◯	◯

Student Confidence After Viewing Pre-Classroom Assigned Videos

9. How confident were you in your ability to perform this exam AFTER reviewing the preparation materials? *

Not Confident	Slightly Confident	Moderately Confident	Very Confident	Extremely confident
◯	◯	◯	◯	◯

10. How likely are you to seek/use other third party videos (ex: Youtube) to supplement the pre classroom assigned videos? *

Very unlikely	Somewhat unlikely	Neutral	Somewhat likely	Very Likely
◯	◯	◯	◯	◯

Student Confidence in Relevant Anatomy

11. How well did the pre-classroom videos explain the relevant anatomy? *

Poor	Fair	Good	Very good	Excellent
◯	◯	◯	◯	◯

12. How comfortable are you with the hip, ankle, and foot Anatomy, as it pertains to the physical exam? *

Not comfortable	Slightly uncomfortable	Moderately comfortable	Very Comfortable	Extremely Comfortable
◯	◯	◯	◯	◯

13. What are the video's **strengths** in explaining the relevant anatomy? *

14. What are the video's **weaknesses** in explaining the relevant anatomy? *

Student Confidence in Performing Special Tests

Special Tests include: Thomas Test, Achilles Tendon Integrity, Intertarsal Neuroma

15. How confident did you feel performing the Hip, Ankle, and Foot special tests after reviewing the preparation materials? *

Not Confident	Slightly confident	Moderately Confident	Very Confident	Extremely Confident
○	○	○	○	○

16. How well did the pre-classroom videos explain the special tests? *

Poor	Fair	Good	Very good	Excellent
○	○	○	○	○

17. How well did the pre-classroom videos explain the pathological mechanisms as it relates to the special tests? *

Poor	Fair	Good	Very good	Excellent
○	○	○	○	○

18. What are the video's **strengths** in preparing you to perform the special tests? *

19. What are the video's **weaknesses** in preparing you to perform the special tests? *

Student Understanding of the Physical Exam

20. **Please rate the following statement:**
The pre-classroom assigned materials gave me more confidence in my ability to perform the hip, ankle, and foot exam. *

Strongly Disagree	Disagree	Neutral	Agree	Strongly agree
◯	◯	◯	◯	◯

21. **Please rate the following statement:**
The pre-classroom assigned materials helped me when practicing the hip, ankle, foot exam. *

Strongly Disagree	Disagree	Neutral	Agree	Strongly agree
◯	◯	◯	◯	◯

22. **Please rate the following statement:**
Please select "Disagree" if you are following along. *

Strongly Disagree	Disagree	Neutral	Agree	Strongly agree
◯	◯	◯	◯	◯

23. How likely are you to watch the pre-classroom assigned materials again to review this exam? (as opposed to other supplemental materials) *

Very unlikely	Somewhat unlikely	Neutral	Somewhat likely	Very Likely
◯	◯	◯	◯	◯

Student Confidence in Using Physical Exam Findings in Differential Diagnosis

24. How confident are you in determining a positive special test versus a negative special test as they pertain to this exam? (Special Tests include: Thomas Test, Achilles Tendon Integrity, Intertarsal Neuroma) *

Not Confident	Slightly Confident	Moderately Confident	Very Confident	Extremely Confident
○	○	○	○	○

25. How confident do you feel using physical exam findings to guide your differential diagnosis (i.e., in your H&P note)? *

Not Confident	Slightly Confident	Moderately Confident	Very Confident	Extremely Confident
○	○	○	○	○

Evaluation of the pre-classroom video

26. What are the strengths of the pre-classroom assigned materials? *

27. What can be improved in the pre-classroom assigned materials? *

28. What worked well in the **format** of the pre-classroom assigned materials? *

29. What could be improved in the **format** of the pre-classroom assigned materials? *

30. What worked well in the **content** of the pre-classroom assigned materials? *

31. What could be improved in the **content** of the pre-classroom assigned materials? *

This content is neither created nor endorsed by Microsoft. The data you submit will be sent to the form owner.

Microsoft Forms
