# Supplementary material for: Effectiveness of video learning materials for musculoskeletal physical examinations in medical skills: a randomized controlled trial
Source: BMC Med Educ. 2026 Jul 17;26:1635. doi: 10.1186/s12909-026-09943-9 (PMC13660170; doi:10.1186/s12909-026-09943-9)
Supplement: Supplementary file 5 [file 12909_2026_9943_MOESM10_ESM.docx]

**Supplemental Figure 9. Knee Examination Likert-scale survey responses for Questions 7–12.**
Student responses for the Knee Examination module are shown for the control and intervention groups. Squares represent the median score, and error bars represent the 25th–75th percentile range. Survey items were scored using a 0–5 Likert scale, with higher scores indicating more favorable responses. Questions shown include perceived quality of special test explanation, perceived quality of pathology explanation, confidence after using the material, use of the video as review material, likelihood of watching the video again, and understanding of positive versus negative tests. Between-group comparisons were performed using the Mann-Whitney U test. Statistical annotations indicate significance levels: ns = not significant. Detailed descriptive and inferential statistics are reported in Table 5.
