# Supplementary material for: Effectiveness of video learning materials for musculoskeletal physical examinations in medical skills: a randomized controlled trial
Source: BMC Med Educ. 2026 Jul 17;26:1635. doi: 10.1186/s12909-026-09943-9 (PMC13660170; doi:10.1186/s12909-026-09943-9)
Supplement: Supplementary file 6 [file 12909_2026_9943_MOESM11_ESM.docx]

**Supplemental Figure 10. Knee Examination Likert-scale survey response for Question 13.**
Student responses for the Knee Examination module are shown for the control and intervention groups. Squares represent the median score, and error bars represent the 25th–75th percentile range. The survey item assessed students’ confidence in performing the knee examination. Responses were scored using a 0–5 Likert scale, with higher scores indicating greater self-reported confidence. Between-group comparison was performed using the Mann-Whitney U test. The statistical annotation indicates no significant difference between groups: ns = not significant. Detailed descriptive and inferential statistics are reported in Table 5.
