# Supplementary material for: Effectiveness of video learning materials for musculoskeletal physical examinations in medical skills: a randomized controlled trial
Source: BMC Med Educ. 2026 Jul 17;26:1635. doi: 10.1186/s12909-026-09943-9 (PMC13660170; doi:10.1186/s12909-026-09943-9)
Supplement: Supplementary file 7 [file 12909_2026_9943_MOESM1_ESM.docx]

**Table S1. Student Free Response Tags For Control vs Intervention Videos**

|  | **Anatomy** | | **Special Test** | | **Video Format** | |
| --- | --- | --- | --- | --- | --- | --- |
| **Tag** | **Control** | **Intervention** | **Control** | **Intervention** | **Control** | **Intervention** |
| Strength | 11 | 26 | 18 | 19 | 16 | 23 |
| Clear Concise | 3 | 2 | 4 | 2 | 2 | 3 |
| Comprehensive | 4 | 4 | 4 | 4 | 0 | 5 |
| Helpful | 8 | 16 | 14 | 10 | 5 | 5 |
| Weakness | 17 | 11 | 11 | 8 | 12 | 10 |
| Lacking Info | 12 | 1 | 4 | 3 | 4 | 2 |
| Suggested to  Add | 9 | 4 | 2 | 3 | 1 | 4 |
| Video Format Issue | 2 | 6 | 4 | 0 | 7 | 4 |
| Video Format Positive | - | - | 2 | 2 | 7 | 11 |
| NA | 1 | 4 | 1 | 10 | 4 | 4 |

**Table S1**. Qualitative data obtained from the free response sections of the surveys were analyzed using Taguette (version XX). Free response questions regarding anatomy yielded the following data: participants in the intervention group reported nearly twice as many “strength” tags (n=26 vs n =11); twice as many “helpful” tags (n=16 vs n= 8); and far less comments about “weakness” (n=11 vs n=17) and “lacking info” (n=1 vs n=12). There were less “suggested to add” tags (n=4 vs n=9) and more “video format issue” tags (n=6 vs n=2) in the intervention group when compared to the control group. Free response questions regarding special tests yielded the following data: participants in the intervention group reported less "helpful" tags (n=10 vs n=14) when compared to control. There were an equal number of “comprehensive” (n=4 and n=4) and “video format positive” (n=2 and n=2) tags across both groups. Free response questions regarding video format showed that the intervention group had more “strength” tags (n=23 vs n=16), “comprehensive” tags (n=5 vs n=0), and “video format positive” tags (n=11 vs n=7).
