# Supplementary material for: Effectiveness of video learning materials for musculoskeletal physical examinations in medical skills: a randomized controlled trial
Source: BMC Med Educ. 2026 Jul 17;26:1635. doi: 10.1186/s12909-026-09943-9 (PMC13660170; doi:10.1186/s12909-026-09943-9)
Supplement: Supplementary file 8 [file 12909_2026_9943_MOESM2_ESM.docx]

**Supplemental Figure 1. Spine/Gait Likert-scale survey responses for Questions 1–6.** Student responses are shown for the control and intervention groups. Squares represent the median score, and error bars represent the 25th–75th percentile range. Survey items were scored using a 0–5 Likert scale, with higher scores indicating more favorable responses. Questions shown include baseline confidence, confidence after reviewing the assigned preparatory material, perceived usefulness of supplemental material, perceived quality of anatomy explanation, comfort with anatomy, and comfort with special tests. Between-group comparisons were performed using the Mann-Whitney U test Statistical annotations indicate significance levels: ns = not significant, (*) p<0.05, (**) p<0.005, (***) p <0.001, (****) p <0.0001
