# Supplementary material for: Effectiveness of video learning materials for musculoskeletal physical examinations in medical skills: a randomized controlled trial
Source: BMC Med Educ. 2026 Jul 17;26:1635. doi: 10.1186/s12909-026-09943-9 (PMC13660170; doi:10.1186/s12909-026-09943-9)
Supplement: Supplementary file 9 [file 12909_2026_9943_MOESM3_ESM.docx]

|  |  |
| --- | --- |

**Supplemental Figure 2. Spine/Gait Likert-scale survey responses for Questions 7–12.**

Student responses are shown for the control and intervention groups. Squares represent the median score, and error bars represent the 25th–75th percentile range. Survey items were scored using a 0–5 Likert scale, with higher scores indicating more favorable responses. Questions shown include perceived clarity of special test explanation, perceived clarity of pathological mechanism explanation, confidence performing the Spurling test, confidence performing the straight leg raise test, confidence in ability to perform the examination, and perceived helpfulness of the video when practicing. Between-group comparisons were performed using the Mann-Whitney U test, with detailed statistical results reported in Table 2. (**) p<0.005, (***) p <0.001, (****) p <0.0001
