# Supplementary material for: Effectiveness of video learning materials for musculoskeletal physical examinations in medical skills: a randomized controlled trial
Source: BMC Med Educ. 2026 Jul 17;26:1635. doi: 10.1186/s12909-026-09943-9 (PMC13660170; doi:10.1186/s12909-026-09943-9)
Supplement: Supplementary file 10 [file 12909_2026_9943_MOESM4_ESM.docx]

**Supplemental Figure 3. Spine/Gait Likert-scale survey responses for Questions 13–14.**

Student responses are shown for the control and intervention groups. Squares represent the median score, and error bars represent the 25th–75th percentile range. Survey items were scored using a 0–5 Likert scale, with higher scores indicating more favorable responses. Question 13 assessed students’ likelihood of watching the video again for review, and Question 14 assessed confidence using physical examination findings to guide diagnosis. Between-group comparisons were performed using the Mann-Whitney U test, with detailed descriptive and inferential statistics reported in Table 2, ns = not significant, (***) p <0.001.
